# Treatment Responses of Cognitive Function and Plasma Asymmetric Dimethylarginine to Atypical Antipsychotic in Patients With Schizophrenia

**DOI:** 10.3389/fpsyt.2018.00733

**Published:** 2019-01-10

**Authors:** Zhi-min Yu, Ying Zhao, Jin-qiong Zhan, Tao Luo, Jian-wen Xiong, Bin Yu, Bo Wei, Yuan-jian Yang

**Affiliations:** ^1^Department of Psychiatry, Jiangxi Mental Hospital/Affiliated Mental Hospital of Nanchang University, Nanchang, China; ^2^Department of Pharmacy, Union Hospital, Tongji Medical College, Huazhong University of Science and Technology, Wuhan, China; ^3^Biological Psychiatry Laboratory, Jiangxi Mental Hospital/Affiliated Mental Hospital of Nanchang University, Nanchang, China

**Keywords:** schizophrenia, cognitive function, asymmetric dimethylarginine (ADMA), atypical antipsychotic, plasma

## Abstract

Cognitive deficits represent a core feature of schizophrenia. Previous studies have demonstrated that plasma asymmetric dimethylarginine (ADMA) was increased in patients with schizophrenia and correlated with cognitive impairments. Atypical antipsychotics can produce cognitive benefits in schizophrenia patients. In this study, we conducted a prospective observation trial to explore whether plasma ADMA may serve as an indicator for evaluating cognitive improvements induced by atypical antipsychotics in patients with schizophrenia. A total of 41 schizophrenia patients with acute exacerbation were enrolled and 29 patients completed this study. These recruited patients were drug-naive or had no exposure to antipsychotics for at least 3 months. Thirty healthy individuals were recruited as a control group. Positive and Negative Syndrome Scale (PANSS) and a neuropsychological battery were used to evaluate schizophrenic symptoms and cognitive function, respectively. Plasma ADMA was measured by high-performance liquid chromatography (HPLC). We found that schizophrenia patients with acute exacerbation had significantly poorer cognitive performances and higher plasma ADMA levels than control individuals (*p* < 0.05). After 2 months of atypical antipsychotic treatment, patients showed significant improvements in processing speed, working memory, attention, and executive function (all *p* < 0.01). Plasma ADMA levels in patients after treatment were significantly decreased compared to baseline (2.42 ± 0.84 vs. 1.55 ± 0.34 μmol/L; *t* = 6.491, *p* < 0.001). Correlation analysis reveals that there is a significant correlation of the decrease in ADMA with improvements in working memory (*r* = −0.413, *p* = 0.026) and attention (*r* = −0.417, *p* = 0.025). Collectively, our results suggest that atypical antipsychotics improve cognitive function in schizophrenia patients with acute exacerbation, in parallel with decreased plasma ADMA levels. Plasma ADMA levels may be an indicator of cognitive recovery in schizophrenia.

## Introduction

Schizophrenia is a chronic mental disorder with a prevalence of ~1% in the population worldwide. Along with positive and negative symptoms, deficits of cognitive function are also deemed as a core feature of schizophrenia (1, 2). A large number of cognitive areas including processing speed, attention, visual memory, verbal learning, working memory, and execute capacity have been reported to be impaired in patients with schizophrenia (3, 4). They are present before the onset of psychosis and may lead to functional disability, including defects in work and social contact, independent living, as well as skill acquirement (5, 6). Thus, the treatment of cognitive deficits has great benefits for the clinical outcome of patients with schizophrenia.

Antipsychotic drug treatment has been recommended to be a key component of schizophrenia treatment algorithms (7). First-generation antipsychotics (FGAs) were discovered in the 1950s and have therapeutic efficacy for positive symptoms, including delusions and hallucinations (8). However, long-term treatment with FGAs can cause a side-effect of cognitive deterioration (9). Since second-generation drugs (SGAs), known as atypical antipsychotics, have been developed and introduced clinically in the 1970s, they have been more frequently used for the management of schizophrenia in recent decades. In addition to positive symptoms, atypical antipsychotics also have significant impacts on negative symptoms and cognitive deficits associated with schizophrenia (9, 10). Numerous double-blind, random-controlled clinical studies have shown that, in compared to FGAs, treatment of atypical antipsychotics could greatly promote cognitive capability in patients with schizophrenia (9–11). Overall cognitive ability and specific domains of cognition such as processing speed, memory, and attention were improved after the usage of atypical antipsychotics (10, 12).

Asymmetric dimethylarginine (ADMA) is an endogenous competitive inhibitor of nitric oxide synthase (NOS). It is physiologically generated during the methylation of protein arginine residues and released during proteolysis (13). Previous studies have demonstrated that plasma ADMA concentrations were increased in patients with schizophrenia relative to healthy individuals, but no association was found between plasma ADMA and the scores of psychiatric rating scales (14–16). We also found that plasma ADMA levels were elevated in schizophrenia patients and increased ADMA correlated with cognitive impairment (17). Zincir et al. reported that treatment with antipsychotics in patients for 2 months could reduce plasma ADMA levels, while no correlation was observed between the improvement of psychiatric symptoms and the change of plasma ADMA (16). However, whether there is a relationship between the reduction of plasma ADMA and cognitive improvement in patients treated with atypical antipsychotics remains unknown.

In this study, we hypothesized that peripheral ADMA levels may be a potential indicator of cognitive recovery in schizophrenia. To test this hypothesis, we investigated whether (1) eight-week atypical antipsychotic monotherapy improves cognition in schizophrenia patients with acute exacerbation; (2) peripheral ADMA is decreased at the endpoint (8th-week) compared to baseline; and (3) improvement of cognition is correlated with change of ADMA levels.

## Materials and Methods

### Subjects

This is an open-label prospective observation study. All patients in this study were recruited from Jiangxi Mental Hospital. The patients of schizophrenia were diagnosed by two trained research psychiatrists using modified sections of the Structured Clinical Interview for Diagnostic and Statistical Manual of Mental Disorders, Fourth Edition (DSM-IV). The age of recruited patients was between 18 and 50 years old and the Positive and Negative Syndrome Scale (PANSS) score need to be between 60 and 120 at the baseline interview. All the patients who were recruited in this study had acute exacerbation for their psychotic symptoms and were admitted into acute ward for assessment and treatment. All these patients were antipsychotic naive or had not taken any antipsychotic drug over the past 3 months. In order to reassure the drug compliance, only patients who stayed well for at least 8-weeks were included in the present study. Healthy individuals were recruited from the local community as controls, matched with the patients by age, gender, education, and body mass index (BMI). All participants were Han Chinese ethnicity.

Patients with neurological disorders, mental retardation, and drug/alcohol abuse or addiction were excluded from this study. Some diseases including metabolic syndrome, diabetes, hyperlipidemia, coronary artery disease, inflammatory disease, liver damage, and renal failure may lead to changes in plasma ADMA (18–23). Thus, besides any additional axis I or axis II DSM-IV diagnosis, the exclusion criteria also included acute or chronic infections, current pregnancy, autoimmune, endocrine, and neoplastic diseases, allergic asthma, and other acute physical disorders, including heart or brain infarction within the past 3 months. Both patient and control groups were assessed with a medical history, physical examination, electrocardiogram, and laboratory tests. They were screened for acute infectious diseases by measuring body temperature, erythrocyte sedimentation rate, serum creatinine, C-reactive protein (CRP), and urinary culture. Subjects with abnormal parameters were excluded, for example urea (Normal range: 2.14~7.85 mmol/L), creatinine (Normal range: 60~130 μmol/L), SGOT (Normal range: 0~40 U/L), SGPT (Normal range: 0~40 U/L), CRP (Normal range: 0~10 mg/L), cholesterol (Normal range: 3.38~6.47 mmol/L), triglycerides (Normal range: 0.4~1.81 mmol/L), systolic and diastolic blood pressure (Normal range: 140/90-90/60 mmHg), and BMI (Normal range: 18.5~24).

All procedures for this study were reviewed and approved by the Institutional Review Board at Jiangxi Mental Hospital. The research was carried out in accordance with the Declaration of Helsinki and other relevant national and international regulations. A written informed consent was obtained from each subject, or his or her legal guardians.

### Evaluations of Clinical Symptom and Cognitive Function

PANSS was used to assess the severity of the psychotic symptoms of patients. The evaluation was conducted by two psychiatrists who had both attended a training session of PANSS use before the study began. The inter-observer correlation coefficient for the PANSS total score was >0.80.

The cognitive function of subjects was measured by a comprehensive battery of neurocognitive tests (17, 24, 25). These tests are commonly used in China, and their clinical reliability and validity have been evaluated in Chinese populations. The battery consists of seven tests and can be grouped into six cognitive domains: processing speed (Trail making test part A: TMT-A; Brief assessment of cognition in schizophrenia-symbol coding: BACS-symbol coding), working memory (Wechsler memory scale-3rd edition-spatial span: WMS-III spatial span), visual memory (Brief visual-spatial memory test-revised: BVMT-R), verbal learning (Hopkins verbal learning test-revised: HVLT-R), attention (Continuous performance test-identical pairs: CPT-IP), and executive function (Stroop color-word test).

TMT-A: In this test, you are given a pencil and a piece of paper with numbered circles, and you are asked to draw a line and connect the circles in the sequence of their numbers. You should draw the lines as quickly and as accurately as you can, since the time taken for the drawing is used for grading.

BACS-symbol coding: In this test, you are given 133 pairs of digits and symbols, and asked to copy the specific symbol as soon as you are shown its paired number. You should do this quickly as the symbols you finish correctly within 120 s are used for the grading.

WMS-III space span: In this test you are faced with a board with ten cubes on it spaced irregularly. An administrator will firstly show you combinations of the cubes in different ways and orders, forward and backward, and then ask you to recall the combinations. On each level of combination, you are given two trials. In the end you are graded in line with your recalled trials.

BVMT-R: In this test you will watch six geometric figures, which will appear three times, each time for 10 s. After that you are asked to draw the figures on a piece of paper in the layout as you watched. The more figures you draw correctly, the better you are rated.

HVLT-R: In this test, you will be presented with twelve Chinese words, which are listed in three categories. The list will be shown three times, followed by a delay time of 25–30 min. After that, you are asked to recall and speak out the words. Your performance is graded in accordance with the words you correctly recalled.

CPT-IP: In this test, you will see digital numbers of 2, 3, and 4 digits flashing on a computer screen. You are asked to immediately click the mouse after you see the same number flash on the screen in a row. In this test, you may hit 90 times in total, and possibly trigger false alarms 90 times in total. You will randomly respond about 270 times in total.

Stroop color-word test: In this test you're presented with three pages: firstly a word page, on which words of colors are black in color, secondly a color page, on which several rows of Xs are in different colors and thirdly a word-color page, on which the same words of colors on the first page are colored using the colors on the second page, though they are not colored in the color of themselves. In each trial you are presentedwith 100 words and you are asked to read them as fast as you can in a cycle time of 45 s. Your correct readings of the words are numbered for your grading.

### Plasma ADMA Level Analysis by High-Performance Liquid Chromatography (HPLC)

Peripheral blood was collected from an antecubital vein between 07:00 and 09:00 a.m. following an overnight fasting at baseline and at the endpoint of the study. The blood samples were centrifuged at 3,000 rpm for 5 min at 4°C. The plasma was separated and stored at −80°C until analysis.

The concentration of plasma ADMA was measured by HPLC as described in our previous study (17). Briefly, we added 5 mg of 5-sulphosalici-licacid (SSA) to 1 mL of plasma and allowed them to mix for 10 min at −20°C. The sample was then centrifuged at 7,000 rpm for 5 min at temperature of 4°C. After that, 10 microliters of the supernatant was mixed with 100 μL of derivatization reagent (prepared by dissolving 10 mg of o-phthaldialdehyde in 0.5 mL of methanol, and adding 2 mL of 0.4 M borate buffer (pH 10.0) and 30 μL of 2-mercaptoethanol). The mixture was subsequently injected into the chromatographic system to determine the level of ADMA. The variability of this method was <7%, and the detection limit of the assay was 0.1 μm.

### Statistical Analysis

Demographic and clinical variables were compared between control and patient groups using Student's *t*-test or analysis of variance (ANOVA) for quantitative variables and chi-squared test for qualitative variables. An analysis of covariance (ANCOVA) was performed to compare the scores of cognitive tests between two groups, controlling for gender, age, years of education, and BMI by using these variables as covariates. To compare the levels of ADMA between the patient and control groups, ANCOVA was performed using gender, age, years of education, and BMI as covariates to minimize the potential effect of these factors on the expression of ADMA levels. A paired *t*-test was performed to analyze plasma concentrations of ADMA between two time points (at baseline and 8th-week after atypical antipsychotic treatment) in the same patients. A Non-parametric test (Kruskal-Wallis test) was used to test whether there was a difference in cognitive function and ADMA level among different atypical antipsychotics at the endpoint. Spearman's correlation analysis was used to examine the correlation between change of cognitive test scores and ADMA level. All statistical tests were two-tailed, and the significance level was set at *p* < 0.05.

## Results

### Demographic and Clinical Characteristics and Cognitive Performance

A total of 41 schizophrenia patients with acute exacerbation consented to the study and received baseline assessments. During the study session, these patients only received one type of atypical antipsychotics. The type of atypical antipsychotic was chosen according to the demographic and clinical symptom characteristics of patients. Patients using clozapine met the criteria of refractory schizophrenia. Benzene hydrochloride was only used when patients developed a marked extrapyramidal response. Of these, 12 patients dropped out for the following reasons: four patients were discharged from hospital before the endpoint of study; five patients did not respond to atypical antipsychotic monotherapy and required a combined treatment with other antipsychotic drugs or modified electric convulsive treatment (MECT); three patients suffered severe impairment of liver function and need to receive liver-protecting therapy. All data reported here are based on the remaining 29 patients who completed this study.

Baseline demographic and clinical characteristics for the patient and control groups are shown in Table 1. There was no significant difference in age, gender, years of education, or BMI between the two groups (all *p* > 0.05). Cognitive tests revealed that schizophrenia patients with acute exacerbation demonstrated significantly poorer cognitive performance than control individuals except for the BVMT-R controlling for age, gender, years of education, and BMI (all *p* < 0.05). We then measured the levels of plasma ADMA in both patient and control groups. The data of 29 patients and 30 healthy controls were included in the analysis of baseline ADMA levels. As previously reported (16, 17), patients with schizophrenia showed higher levels of plasma ADMA relative to the controls (2.42 ± 0.84 vs. 1.41 ± 0.14 μmol/L; *F* = 42.947, *p* < 0.001). ANCOVA analysis revealed that the difference between two groups still existed (*F* = 10.098, *p* < 0.001) when age, gender, years of education, and BMI were used as covariates.

**Table 1:** Baseline demographic and clinical characteristics for patient and control groups.

	**Patients**	**Controls**	***F/χ*2**	***p***
Age (years)	31.17 ± 8.19	32.03 ± 8.25	0.162	0.689
Gender (M/F)	14/15	16/14	0.151	0.797
Education (years)	9.79 ± 4.90	11.73 ± 5.22	2.163	0.147
BMI (kg/m^2^)	21.43 ± 1.78	20.93 ± 1.87	1.110	0.297
Duration of illness (years)	6.82 ± 5.56	NA		
**PANSS**				
Total scores	80.14 ± 8.21	NA		
Positive subscore	23.10 ± 4.72	NA		
Negative subscore	14.14 ± 5.13	NA		
General psychopathology	43.90 ± 4.15	NA		
**COGNITIVE FUNCTION**				
TMT-A	69.48 ± 23.37	40.37 ± 10.33	7.511	<0.001
BACS-SC	31.52 ± 11.03	65.63 ± 5.93	43.472	<0.001
WMS-III-SS	13.92 ± 2.86	17.62 ± 3.31	5.311	<0.001
HVLT-R	20.17 ± 6.72	26.27 ± 5.37	3.247	0.012
BVMT-R	21.41 ± 5.20	26.70 ± 9.29	2.314	0.066
CPT-IP	1.34 ± 0.88	3.46 ± 1.03	14.175	<0.001
Stroop word score	52.79 ± 13.98	85.87 ± 8.15	26.982	<0.001
Stroop color score	31.24 ± 14.29	50.57 ± 8.93	7.633	<0.001
Stroop color-word score	19.72 ± 11.14	37.10 ± 8.05	10.449	<0.001
Plasma ADMA (μmol/L)	2.42 ± 0.84	1.41 ± 0.14	10.098	<0.001

*BMI, body mass index; PANSS, Positive and Negative Syndrome Scale; TMT-A, trail making task part A; BACS-SC, brief assessment of cognition in schizophrenia-symbol coding; CPT-IP, continuous performance test-identical pairs; WMS-III-SS, Wechsler memory scale-3rd edition-spatial span; HVLT-R, Hopkins verbal learning test-revised; BVMT-R, brief visual-spatial memory test-revised. NA, not applicable*.

### Clinical Efficacy and Neurocognitive Effect of Atypical Antipsychotic in Patients

The primary endpoint was the change from baseline to 8-weeks in PANSS and cognitive test scores. The number of patients treated with risperidone, olanzapine, clozapine, and aripiprazole was 10, 5, 7, and 7, respectively. The final median dose of risperidone, olanzapine, clozapine, and aripiprazole was 3.12 ± 1.05, 16.27 ± 5.33, 292.75 ± 115.63, and 14.66 ± 3.81 mg/day, respectively. Table 2 shows the clinical efficacy and neurocognitive effect of atypical antipsychotics in schizophrenia patients with acute exacerbation. A significant improvement in PANSS total score and each subscore was observed when the patients completed this study (Paired *t*-tests; all *p* < 0.01). According to the proposed criteria for symptomatic remission in patients with schizophrenia (26), the rate of symptomatic remission at the endpoint was 82.75% (24/29). Additionally, patients given an 8-week period of atypical antipsychotic treatment showed significant improvements in the test scores of TAM-A, BACS-SC, WMS-III, CPT-IP, and Stroop color-word test (all *p* < 0.01). Furthermore, Kruskal-Wallis test showed that there was no difference in PANSS total score, each subscore, or the cognitive tests mentioned above, among risperidone, olanzapine, clozapine, and aripiprazole-treated groups at the endpoint (*p* > 0.05).

**Table 2:** Comparison of PANSS and cognitive test scores between baseline and endpoint in patients.

	**Baseline**	**Endpoint**	***t***	***p***
**PANSS**				
Total scores	80.14 ± 8.21	36.14 ± 9.27	29.653	<0.001
Positive subscore	23.10 ± 4.72	7.86 ± 2.29	19.921	<0.001
Negative subscore	14.14 ± 5.13	7.72 ± 1.23	9.149	<0.001
General psychopathology	43.90 ± 4.15	20.55 ± 6.09	18.517	<0.001
**COGNITIVE FUNCTION**				
TMT-A	69.48 ± 23.37	51.83 ± 17.06	5.324	<0.001
BACS-SC	31.52 ± 11.03	43.21 ± 12.41	−4.393	<0.001
WMS-III-SS	13.92 ± 2.86	17.08 ± 3.52	−5.556	<0.001
HVLT-R	20.17 ± 6.72	23.14 ± 11.13	−1.995	0.056
BVMT-R	21.41 ± 5.20	23.66 ± 8.01	−1.641	0.112
CPT-IP	1.34 ± 0.88	1.87 ± 0.63	−2.809	0.009
Stroop word score	52.79 ± 13.98	74.31 ± 12.65	−7.093	<0.001
Stroop color score	31.24 ± 14.29	44.28 ± 12.48	−4.379	<0.001
Stroop color-word score	19.72 ± 11.14	29.52 ± 12.69	−4.937	<0.001

### Plasma ADMA Level After Atypical Antipsychotic Treatment

**Figure 1:**
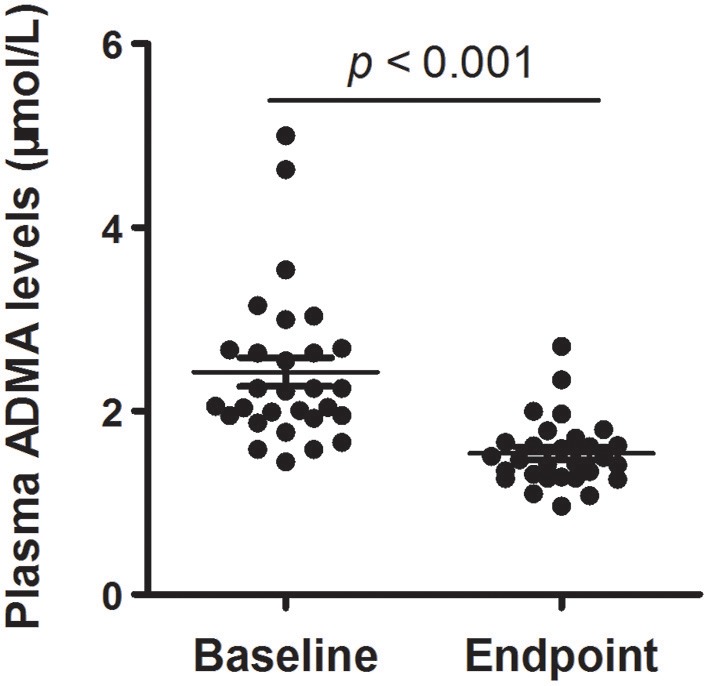
Plasma ADMA levels of schizophrenia patients with acute exacerbation at baseline and endpoint (8th-week). Scatter plot for the comparison of plasma ADMA levels at baseline and endpoint. The sample means are indicated by the black bars.

Together with clinical improvement, plasma ADMA levels after 8-week atypical antipsychotic monotherapy were also significantly decreased compared with baseline (2.42 ± 0.84 vs. 1.55 ± 0.34 μmol/L; *t* = 6.491, *p* < 0.001) (Figure 1). There was no significant difference in plasma ADMA levels between the patients after atypical antipsychotic treatment and the controls (1.55 ± 0.34 vs. 1.41 ± 0.14 μmol/L; *p* > 0.05). Furthermore, all these atypical antipsychotics could lead to a significant decrease in the levels of plasma ADMA (*p* < 0.05), and there was no significant difference between groups in plasma ADMA levels at the study endpoint (Kruskal-Wallis test, χ^2^ = 4.255, *p* = 0.235) (Table 3).

**Table 3:** Plasma ADMA levels before and after treatment.

	**Baseline ADMA (μmol/L)**	**Endpoint ADMA (μmol/L)**	***t***	***p***
Risperidone (*n* = 10)	2.08 ± 0.48	1.46 ± 0.29	3.946	0.003
Olanzapine (*n* = 5)	2.35 ± 0.68	1.42 ± 0.13	2.857	0.046
Clozapine (*n* = 7)	2.80 ± 1.06	1.73 ± 0.57	3.965	0.007
Aripiprazole (*n* = 7)	2.59 ± 1.03	1.61 ± 0.01	2.571	0.042
χ2	3.102	4.255		
*p*	0.376	0.235		

### Relationship Between Cognitive Improvement and Change of Plasma ADMA Levels

**Figure 2:**
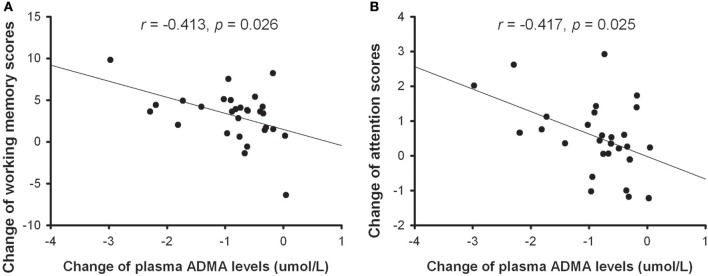
The correlation between the change of plasma ADMA levels and improvement of working memory **(A)** or attention **(B)** in patients with atypical antipsychotic treatment.

Spearman's correlation analysis was conducted to explore whether the change in plasma ADMA levels was correlated with clinical improvements. Similar to a previous study (16), we did not find any relationship between the reduction of plasma ADMA and the change of PANSS total score (*r* = 0.028, *p* = 0.886), positive subscore (*r* = −0.021, *p* = 0.913), negative subscore (*r* = −0.044, *p* = 0.821), or general psychopathology (*r* = 0.055, *p* = 0.776). Then we further analyzed the relationship between the change of plasma ADMA levels and cognitive improvement. As shown in Figure 2, there is a significant correlation between the decrease of ADMA and the change in WMS-III (*r* = −0.413, *p* = 0.026) or CPT subscore (*r* = −0.417, *p* = 0.025), indicating that the decrease in plasma ADMA was correlated with the improvements of working memory and attention in patients treated with atypical antipsychotic.

## Discussion

This is a prospective, open-label, 8-week observational study, aiming to explore the efficacy of atypical antipsychotics on cognitive deficits in patients with schizophrenia. We found that schizophrenia patients with acute exacerbation displayed multi-faceted cognitive impairments in comparison with healthy individuals. Biologically, plasma levels of ADMA were much higher in patients than in controls. 8-week treatment with atypical antipsychotic significantly improved psychiatric symptoms and cognitive function, as well as leading to decreased plasma ADMA levels in patients. The decrease in ADMA levels is significantly associated with cognitive improvement of patients.

Cognitive deficits are a core feature of schizophrenia. Consistent with previous reports (17, 24, 25), our present study reveals that schizophrenia patients with acute exacerbation exhibited a wide range of cognitive impairments across multiple domains including processing speed, working memory, attention, verbal learning, and executive capability. Treatment with risperidone, olanzapine or aripiprazole could improve specific cognitive domains with similar global clinical efficacy in schizophrenia patients (10, 12). Results from animal experiments also showed that these atypical antipsychotics had positive benefits for cognitive deficits in a rat model of schizophrenia (27–29). In the present study, the recruited patients received medication of risperidone, olanzapine, clozapine or aripiprazole, and they showed significant improvements in cognitive domains of processing speed, working memory, attention, and executive function after 2 months of treatment. These results demonstrate the efficacy of atypical antipsychotics in cognitive impairments of schizophrenia patients with acute exacerbation.

Nitric oxide (NO) is a gas messenger that exerts multiple biological effects in the central nervous system. Deficits of NO signaling are shown to be linked with the pathogenesis of schizophrenia (30, 31). For example, Reif et al. reported that regulatory polymorphisms of NOS contributed to the genetic risk for schizophrenia (32). A decrease in nitrergic neurons was found in striatum of schizophrenia patients (33). The levels of NO and its metabolites were decreased in serum or plasma of patients with schizophrenia (14, 34). As an endogenous competitive inhibitor of NOS, ADMA was increased in the plasma of schizophrenia patients (16, 17). Treating schizophrenia patients with antipsychotic for 2 months led to a significant reduction in plasma ADMA levels (16). Consistent with this report, our present study showed that the levels of plasma ADMA in patients after 8-week atypical antipsychotic treatment were significantly decreased compared to that in controls. No difference was found in ADMA levels among patients-treated with risperidone, olanzapine, clozapine or aripiprazole at the study endpoint, demonstrating similar efficacy of these antipsychotics in the regulation of plasma ADMA levels in patients.

NO plays an essential role in synaptic plasticity and cognition (35, 36). Inhibition of endogenous NO generation impairs synaptic plasticity and learning and memory (37–39). As an endogenous inhibitor of NOS, ADMA decreases the levels of NO by inhibition of NOS activity (13). Our previous study found that increased plasma ADMA was associated with cognitive deficits in patients with schizophrenia (17). The results of our present study showed that atypical antipsychotic treatment could significantly decrease plasma ADMA levels and parallelly improve cognitive function in schizophrenia patients with acute exacerbation. A series of studies have demonstrated the efficacy of NO on cognitive impairments in animal models of schizophrenia (31). Specifically, central supplement of S-nitroso-N-acetylpenicillamine (SNAP), an NO donor, attenuated the impaired spatial memory in MK-801-treated mice (40). Intraperitoneal injection of GT 1061, a novel nitrate that enhance NO release, reversed MK-801-induced reversal learning deficits in rats (41). Treatment with sodium nitroprusside attenuated the attention impairment produced by amphetamine in the prepulse inhibition test in mice (42). In view of the role of NO in schizophrenia-related cognitive deficits and inhibition of endogenous ADMA on NO signaling, we postulated that atypical antipsychotic may exert a beneficial effect on cognitive function by upregulating NO signaling in patients with schizophrenia. However, further investigations are needed to address this postulation.

An interesting finding of this study is that improvement of cognitive function was associated with decreased ADMA levels, in patients treated with atypical antipsychotic. This finding indicates that the plasma ADMA level may be a potential indicator for evaluating the outcome for cognitive deficits after atypical antipsychotic treatment. Although underproduction of NO has been shown to contribute to the pathology of schizophrenia and decreased levels of plasma NO have been found in schizophrenia patients (14, 31), NO is not suitable to be a biomarker because it has a very short period of existence in plasma. ADMA levels are relatively stable and can be accurately measured in plasma (13). Thus, it may be more suitable to serve as a peripheral biomarker than NO. However, due to the small sample size of this study, further large-scale clinical studies are needed to confirm the value of plasma ADMA as an indicator for cognitive evaluation in schizophrenia.

Despite the suggestive results, some limitations of the present study should be noted. First, this is an open-label prospective observational study and selection bias may have been introduced. Generalization of these findings beyond the population should be cautious. Second, we measured the level of ADMA in plasma. Whether plasma ADMA can reflect a similar change in the brain is still uncertain. Third, each type of atypical antipsychotics may produce cognitive benefits in specific cognitive domains (12). However, the sample size of this study is small and limits further analysis of the correlation between improvement of specific cognitive domain and ADMA decrease stratified by type of atypical antipsychotics. Thus, large-scale clinical studies are needed to replicate and verify these findings. Fourth, Spearman's correlation analysis reveals a relationship between cognitive improvement and ADMA decrease in patients with schizophrenia. However, a causal relationship between the two variables cannot be drawn. Further research using animal experiments is needed to address this issue. Fifth, the symptomatic remission rate found in our present study (82.75%) exceeded reported rates (16 to 78%) (43). Perhaps the type of antipsychotics and the criteria for data collection could partially explain the difference. All the patients recruited in this study were treated with atypical antipsychotics, which might improve the treatment compliance of patients and thus resulted in a higher remission rate. In addition, as patients who did not respond to atypical antipsychotic monotherapy and required combined treatment with other antipsychotic or MECT were considered as dropout and were not included in the data analysis, this might also have contributed to the high statistical value for theremission rate. The sample characteristics of this study limit the generalization of the results. Collection of data from patients treated with different types of antipsychotics (both typical and atypical antipsychotics), as well as patients with combined antipsychotic treatment, is needed to obtain a more exact value for the remission rate. Finally, we did not examine the pathway of ADMA/NOS/NO in the present study. NOS activity and nitrate and nitrite levels should be measured to verify that there is a corresponding change in NO levels.

In conclusion, our present study involved a prospective, open-label, 8-week observational trial to explore the effects of atypical antipsychotics on cognitive function and plasma ADMA levels in patients with schizophrenia. The results showed that atypical antipsychotic treatment in schizophrenia patients with acute exacerbation improved psychiatric symptoms and cognitive function, particularly working memory and attention, in parallel with decreased plasma ADMA levels. The change in ADMA was negatively correlated with the improvement of cognition, suggesting that plasma ADMA may be a potential indicator of cognitive recovery in schizophrenia.

## Author Contributions

ZY, YZ, JZ, TL, JX, and BY were responsible for the clinical data collection and laboratory experiments. YY and BW were responsible for the study design, statistical analysis, and manuscript preparation. All authors have contributed to and approved the final manuscript.

### Conflict of Interest Statement

The authors declare that the research was conducted in the absence of any commercial or financial relationships that could be construed as a potential conflict of interest.
